# Habitual tea drinking modulates brain efficiency: evidence from brain connectivity evaluation

**DOI:** 10.18632/aging.102023

**Published:** 2019-06-14

**Authors:** Junhua Li, Rafael Romero-Garcia, John Suckling, Lei Feng

**Affiliations:** 1 Laboratory for Brain-Bionic Intelligence and Computational Neuroscience, Wuyi University, Jiangmen, China; 2 Centre for Life Sciences, National University of Singapore, Singapore; 3 School of Computer Science and Electronic Engineering, University of Essex, Colchester, United Kingdom; 4 Brain Mapping Unit, Department of Psychiatry, University of Cambridge, Herchel Smith for Brain and Mind Sciences, Cambridge, United Kingdom; 5 Department of Psychological Medicine, Yong Loo Lin School of Medicine, National University of Singapore, Singapore

**Keywords:** tea drinking, brain efficiency, fMRI, DTI, default mode network, hemispheric asymmetry

## Abstract

The majority of tea studies have relied on neuropsychological measures, and much fewer on neuroimaging measures, especially for interregional connections. To date, there has been no exploration of the effect of tea on system-level brain networks. We recruited healthy older participants to two groups according to their history of tea drinking frequency and investigated both functional and structural networks to reveal the role of tea drinking on brain organization. The results showed that tea drinking gave rise to the more efficient structural organization, but had no significant beneficial effect on the global functional organization. The suppression of hemispheric asymmetry in the structural connectivity network was observed as a result of tea drinking. We did not observe any significant effects of tea drinking on the hemispheric asymmetry of the functional connectivity network. In addition, functional connectivity strength within the default mode network (DMN) was greater for the tea-drinking group, and coexistence of increasing and decreasing connective strengths was observed in the structural connectivity of the DMN. Our study offers the first evidence of the positive contribution of tea drinking to brain structure and suggests a protective effect on age-related decline in brain organisation.

## INTRODUCTION

Tea has been a popular beverage since antiquity, with records referring to consumption dating back to the dynasty of Shen Nong (approximately 2700 BC) in China [1]. Tea is consumed in diverse ways, with brewed tea and products with a tea ingredient extremely prevalent in Asia, especially in China and Japan. It also is more fashionable than ever in western countries. A growing literature has demonstrated that tea intake is beneficial to human health, including mood improvement (e.g., anti-stress) [2–4], risk reduction of cognitive decline [5–8], cardiovascular disease prevention [9], lower cancer incidence [10, 11], reduced mortality [12, 13]. These benefits of tea are derived primarily from the effects of its constituents: catechin, L-theanine, and caffeine. In both animal and human studies [14–16], catechin has been found to be beneficial to cognitive health, showing enhancements in memory recognition and working memory performance compared to the intake of placebo [14]. Kimura et al. discovered that L-theanine plays a positive role in anti-stress by reducing stress-induced heart rate and salivary immunoglobulin A (s-lgA) during a stressed mental arithmetic task [3]. The beneficial effect of caffeine on cognitive functioning was reported at least two decades ago [17] and replicated by recent studies [18, 19]. Although individual constituents of tea have been related to the roles of maintaining cognitive abilities and preventing cognitive decline, a study with behavioural and neurophysiological measures showed that there was a degraded effect or no effect when a constituent was administered alone and a significant effect was observed only when constituents were combined [20]. The superior effect of the constituent combination was also demonstrated in a comparative experiment [21] that suggested that tea itself should be administered instead of tea extracts; a review of tea effects on the prevention of Alzheimer’s disease (AD) [22], found that the neuroprotective role of herbal tea was apparent in eight out of nine studies.

It is worth noting that the majority of studies thus far have evaluated tea effects from the perspective of neurocognitive and neuropsychological measures, with direct measurement of brain structure or function less-well represented in the extant literature (see a recent summary in [23]). In a double-blind, placebo-controlled, crossover study with near-infrared spectroscopy measure, cerebral blood flow in the frontal cortex was reduced by oral tea administration [24]. This change of regional brain activity was also observed by EEG in a study, showing that higher theta, alpha, and beta oscillations were associated with tea consumption in the frontal and medial frontal gyri [25]. These studies focusing on brain regional alterations did not ascertain tea effects on interregional interactions at the level of the entire brain.

Graph theoretical analysis is a suitable and effective tool to gain insights into brain interregional interactions and has been widely utilized in diverse investigations involving both patients and healthy people [26–28]. To date, only two published papers have investigated the association between functional connectivity and tea compounds in only a few task-related regions selected a priori [29, 30], leaving the large-scale networks of the brain unexplored.

In this study, we comprehensively explored brain connectivity with both global and regional metrics derived from structural and functional imaging to unveil putative differential connectivity organizations between tea drinking group and non-tea drinking group. In addition, we focused on interregional connectivity within the default mode network (DMN) because previous studies have suggested that it is predominantly involved in cognitive disease [31] and normal ageing [32]. Moreover, according to our prior investigation of hemispheric asymmetry, leftward asymmetry in structural connectivity is associated with brain ageing [33]. Therefore, hemispheric asymmetries in connectivity were also included to test the effects of tea drinking. We hypothesized that: (1) habitual tea drinking has positive effects on brain organization and gives rise to greater efficiency in functional and structural connectivities; (2) tea intake leads to less leftward asymmetry in structural connectivity; (3) tea drinking is associated with connective strength alterations of functional and structural connectivities in the DMN.

## RESULTS

### Demographic information, neuropsychological and cognitive measures

**Figure 1:**
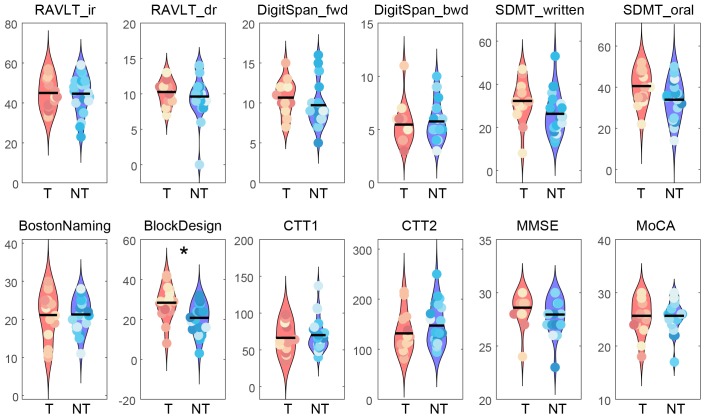
**Comparisons of neuropsychological and cognitive measures between the tea drinking group (T) and non-tea drinking group (NT).** A non-parametric permutation test was used to evaluate the significance level in group differences by permuting 10,000 times (* corrected p<0.05). Abbreviations: RAVLT_ir, Rey Auditory Verbal Learning Test with immediate recall; RAVLT_dr, Rey Auditory Verbal Learning Test with delayed recall; DigitSpan_fwd, forward Digit Span; DigitSpan_bwd, backward Digit Span; SDMT_written, Symbol Digit Modalities Test by written response; SDMT_oral, Symbol Digit Modalities Test by oral response; BostonNaming, Boston Naming Test; BlockDesign, Block Design tests from the Wechsler Adults Intelligence Scale (WAIS-III); CTT1, Color Trials Test 1; CTT2, Color Trials Test 2; MMSE, Mini-Mental State Examination, MoCA, Montreal Cognitive Assessment.

Demographic information is listed in Table 1. There was no significant difference between the tea-drinking and the non-tea drinking groups in age (t_34_=0.92, p>0.05) and years of education (t_34_=0.95, p>0.05) using a two-tailed, two-sample t-test. A Chi-square test did not demonstrate any significant differences between the groups in the ratio of male to female participants (χ^2^(1)=1.85, p>0.05), or the ratio of left-handedness to right-handedness (χ^2^(1)=0.73, p>0.05). Coffee consumption was not significantly different between the groups (t_34_=-0.48, p>0.05). In the comparisons of neuropsychological and cognitive measures, one out of 12 measures were significantly different between the tea-drinking and the non-tea drinking groups according to the permutation test (see Figure 1). Higher performance was observed for the tea-drinking group in the Block Design test (corrected p=0.042).

**Table 1:** Demographic information of participants in this study.

	**Tea drinking group**	**Non-tea drinking group**	**Statistic**	**Significance**
Age (Mean±STD)	70.27±5.52	71.71±3.98	t(34)=-0.92	p=0.37a
Years of Education (Mean±STD)	6.00±3.96	4.81±3.50	t(34)=0.95	p=0.35a
Gender (Male/Female)	4/11	2/19	χ2(1)=1.85	p=0.17b
Handedness (Left/Right)	0/15	1/20	χ2(1)=0.73	p=0.39b

^a^ Two-tailed two-sample t-test

^b^ Chi-square test

### Graph theoretical analysis

**Figure 2:**
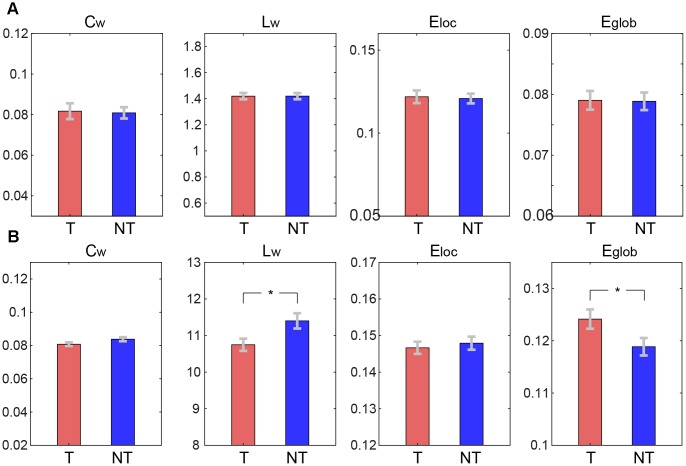
**Comparisons of global graph theoretical metrics between tea drinking (T) and non-tea drinking groups (NT).** (**A**) Metrics computed on the functional connectivity network. (**B**) Metrics calculated on the structural connectivity network. Asterisks represent significance level obtained by permutation test (* corrected p< 0.05).

**Figure 3:**
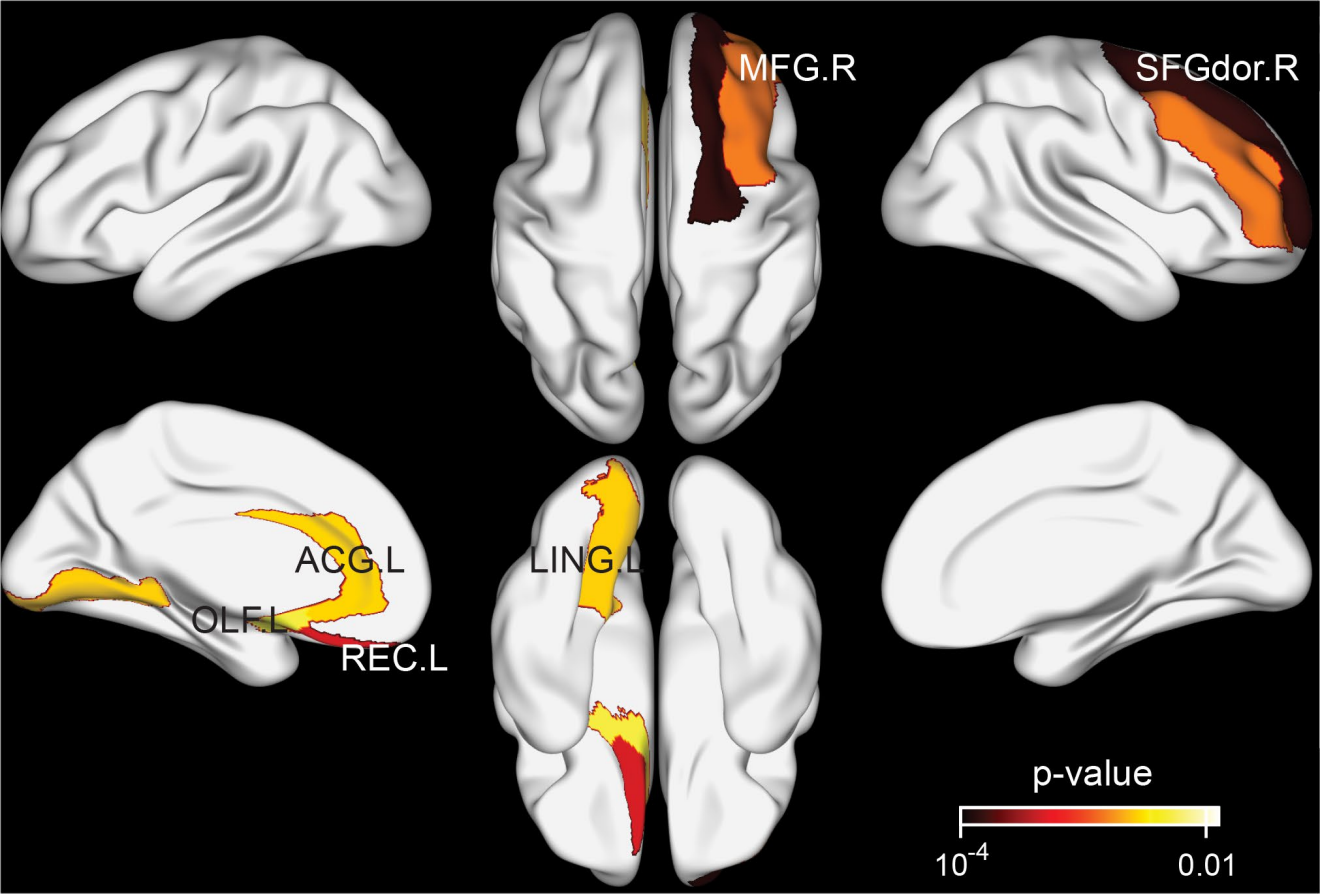
**Brain regions exhibiting significant differences in structural nodal efficiency between the tea drinking group and the non-tea drinking group at the significance level of 0.01 (uncorrected) statistical evaluated by a permutation test.**

No significant differences were found between the tea-drinking group and the non-tea drinking group in global functional network measures C_w_, L_w_, E_loc_, and E_glob_ (p>0.05) (see Figure 2). In the structural network, significantly lower L_w_ (corrected p=0.044) and significantly higher E_glob_ (corrected p=0.044) were observed in the tea-drinking group, whilst no significant differences were found with C_w_ and E_loc_ (p>0.05). Similarly, we did not observe any significant differences in regional measures between the groups in the functional network, but we found 6 regions which were significantly different in the structural network (uncorrected p<0.01): right superior frontal gyrus (dorsal) [SFGdor.R], right middle frontal gyrus [MFG.R], left olfactory [OLF.L], left gyrus rectus [REC.L], left anterior cingulate and paracingulate gyri [ACG.L], and left lingual gyrus [LING.L], which primarily reside in the frontal cortex (see Figure 3).

### Hemispheric asymmetry

**Figure 4:**
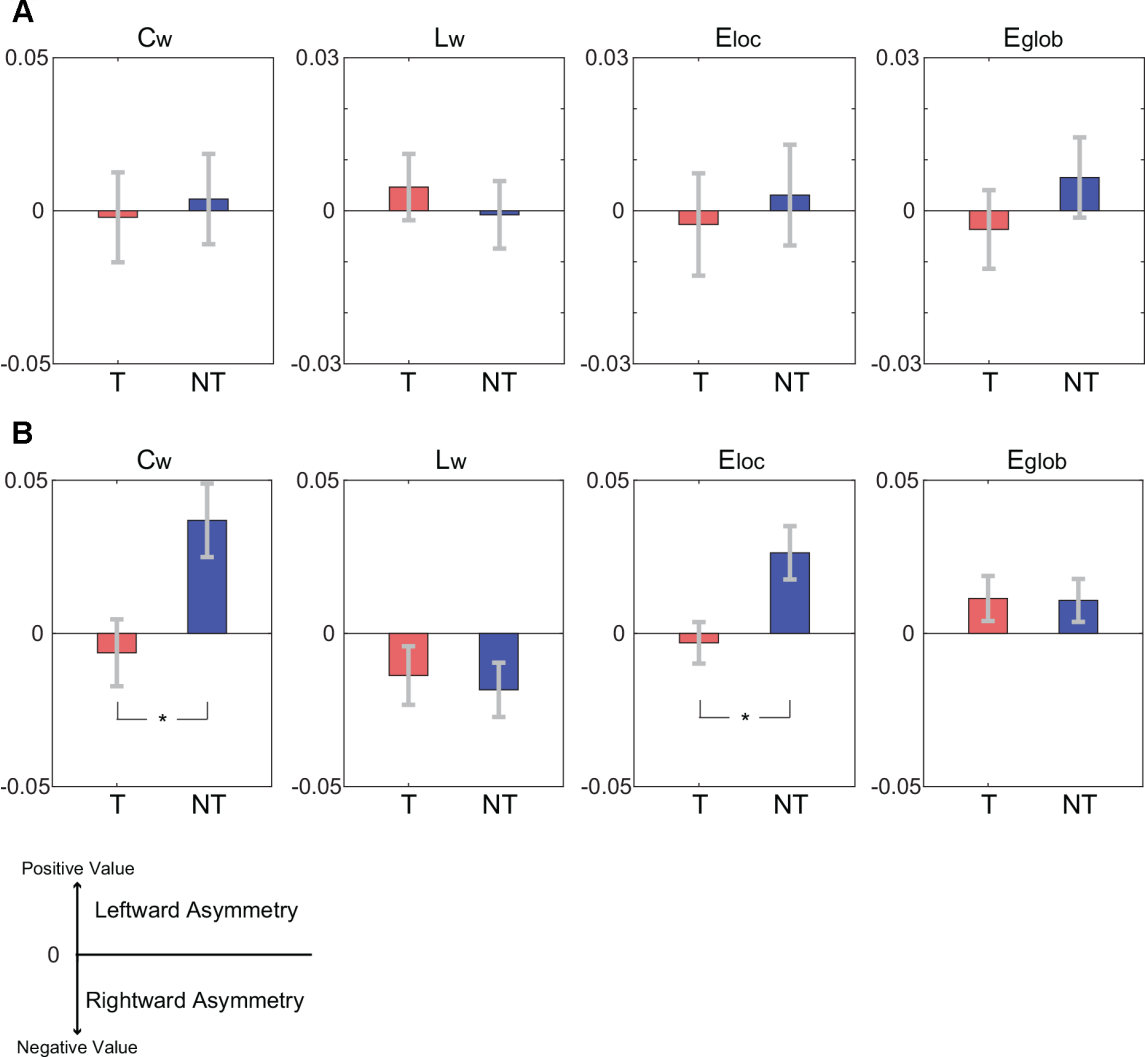
**Comparisons of hemispheric asymmetries of global graph theoretical metrics between the tea-drinking group (denoted by T) and the non-tea drinking group (denoted by NT).** A positive value in the hemispheric asymmetry indicates the leftward hemispheric asymmetry while a negative value indicates the rightward hemispheric asymmetry. Asterisks represent significance level obtained by permutation test (* corrected p< 0.05). (**A**) Hemispheric asymmetries of global graph theoretical metrics for functional connectivity network. (**B**) Hemispheric asymmetries of global graph theoretical metrics for structural connectivity network.

The comparisons of hemispheric asymmetries of global graph theoretical metrics between the tea-drinking group and the non-tea drinking group are illustrated in Figure 4. Hemispheric asymmetries in C_w_ and E_loc_ were significantly different between the groups in the structural network (both, corrected p=0.018), exhibiting greater asymmetry between hemispheres in the non-tea drinking group. Both L_w_ and E_glob_ in the structural network and all metrics in the functional network were not significant in terms of asymmetry.

### Default mode network

**Figure 5:**
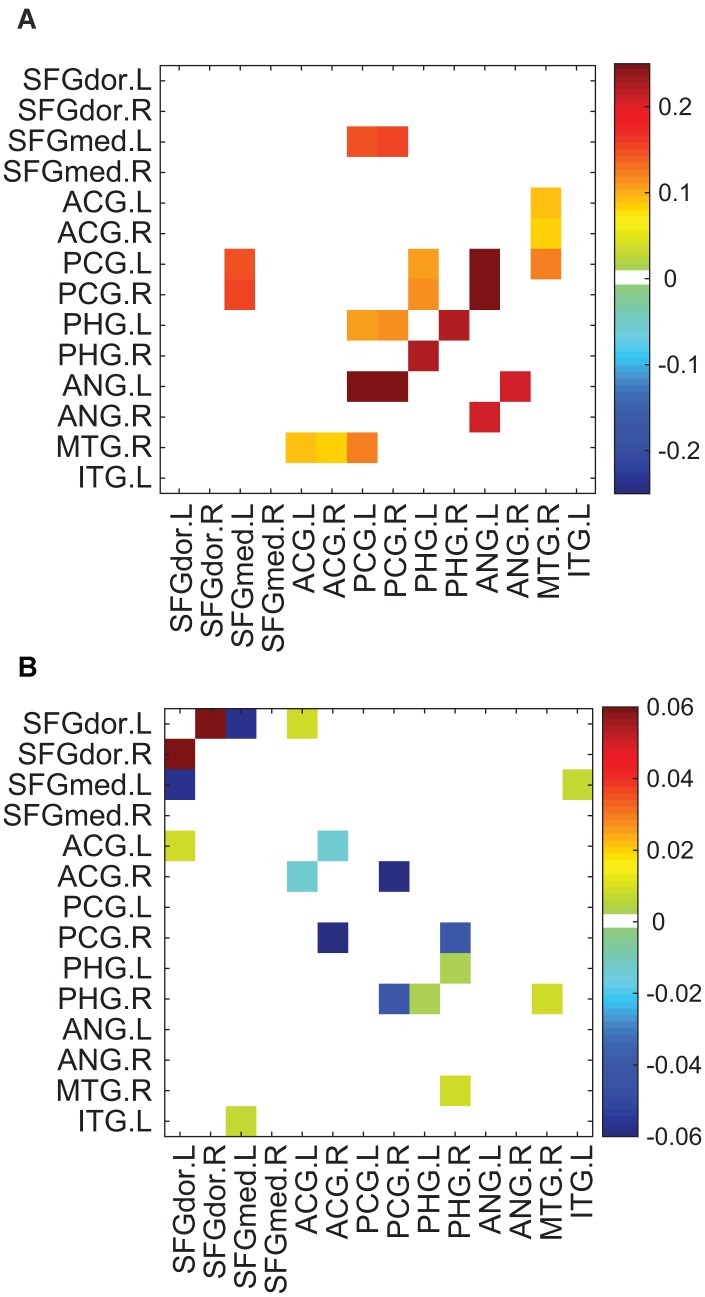
**Connections with significantly different strengths between the tea drinking group and the non-tea drinking group within the default mode network. Significance was established by setting uncorrected p<0.05 with the permutation test.** (**A**) Differences of connective strengths between the groups (tea drinking group minus non-tea drinking group) for significant connections in the functional network. (**B**) Differences of connective strengths between the groups for significant connections in the structural network.

The exploration of strengths of connections within the default mode network revealed consistently increased strength of functional connectivity and the coexistence of increased and decreased strengths for the structural connectivity in the tea drinking group compared to the non-tea drinking group (See Figure 5). Specifically, eleven functional connections exhibited a significant enhancement in strength in the tea drinking group, in which the PCG, PHG, ANG were predominantly involved (see Figure 5A). There was no functional connection with a strength that was significantly decreased for the tea drinking group relative to the non-tea drinking group. Unlike in functional connections, strengths in the structural connections showed a pattern of both increases and decreases (see Figure 5B). The number of structural connections with significantly increased strength was comparable to that of structural connections with significantly decreased strength.

## DISCUSSIONS

In this study, we comprehensively investigated both functional and structural networks from the perspectives of global and regional properties for the effect of habitual tea drinking on the human brain. The observations in this study partially support the hypothesis that tea drinking has positive effects on brain organization and gives rise to greater efficiency in functional and structural connectivities due to increased global network efficiency found in the brain structure of tea drinkers, but no significant enhancement in functional connectivity. As hypothesised, tea drinking leads to less leftward asymmetry in structural connectivity between hemispheres. In addition, we found that functional connectivity within the DMN was higher for the tea drinking group. A coexistence of increasing and decreasing connectivity strengths in the structural DMN was unveiled. These findings are consistent with the hypothesis that there is an association between tea drinking and connective strength alterations.

A large number of studies have suggested that the reduction of interregional connectivity is associated with brain ageing by means of diverse data analytics such as seed-based connectivity, predefined regions interconnections, and tractographic connectivity [34–39], which implies that a slower ageing brain should retain more connections between regions and milder disruption in connectivity resulting in higher efficiency for communications and information exchange between regions. Here, higher structural network efficiency was found in older adults who had habitual tea drinking. Relative to the non-tea drinking group, the tea drinking group had less topological distance between brain regions and more efficient interregional connectivity. This supports the hypothesis that the positive effects of habitual tea drinking are exerted on brain organization by preventing interregional connections from becoming disrupted. In addition to the significant effect of tea drinking on whole brain organization, a regional effect was also observed in this study, showing that tea intake resulted in higher nodal efficiency. The majority of these regions reside in the frontal cortex, which is in agreement with the previous finding that the frontal region is involved in age-related alterations of structural connectivity [34].

In contrast to structural connectivity, functional connectivity did not exhibit any significant difference in both global graph theoretical metrics and regional metrics. Habitual tea drinking did not give rise to observable changes in brain functional connectivity relative to non-tea drinking. If differences in functional connectivity do exist, then they may be masked by the well-known compensatory mechanism [40]. The loss of structural connectivity might be compensated for by greater functional activity so as to preserve equivalent function corresponding to intact structural connectivity [41]. Another possible explanation is that abnormalities in functional connectivity are too subtle to manifest in measures of connectivity efficiency. These two possible explanations are compatible; that is, a subtle change was residual after the compensation counteracting detrimental alterations. However, the effect of compensation is finite [40] and cannot always completely offset the decrement in cognitive performance. This was supported by the results of the neuropsychological measures. Greater visuospatial functioning was observed in older adults with tea drinking compared to older adults without tea drinking based on the test of Block Design, while no significant difference was found in the other measures. Our observation suggests that tea consumption might provide a beneficial effect that cannot be derived from compensation to facilitate the implementation of cognitive tasks.

In the comparison of hemispheric asymmetry between the tea drinking group and non-tea drinking group, the suppression of hemispheric asymmetry in structural connectivity was associated with tea drinking, tending to be more symmetric in structural connectivity. Specifically, the non-tea drinking group exhibited significantly leftward asymmetry in the clustering coefficient and local efficiency, and thus the segregation of connectivity networks was distinct between brain hemispheres. This hemispheric asymmetry in structural connectivity has been associated with brain ageing [42]. Moreover, leftward asymmetry in structural connectivity was found in healthy older adults [33]. Collectively, previous studies have suggested a U-shaped developmental trajectory in hemispheric asymmetry from leftward asymmetry to rightward asymmetry to leftward asymmetry again across the lifespan from childhood to middle age to old age [33, 42–45]. Taken together, the suppression of leftward asymmetry in structural connectivity suggests that tea intake could slow age-related alterations towards leftward asymmetry and retain a pattern more similar to that of the middle age (i.e., rightward asymmetry). In addition, we found six significantly differential regions between the groups, which showed greater efficiency in the tea-drinking group. These regions included MFG, SFGdor, and ACG, which were also found to have an age-related association in a previous study [33]. This suggests that tea plays a beneficial role in the preservation of efficiency between brain regions. The differentiation of hemispheric asymmetry between the tea-drinking group and the non-tea drinking group might underpin significant manifestations between the groups in visuospatial functioning and information processing, as the right hemisphere is more specialized for visuospatial processing [46]. Similar to the results of the whole-cerebrum functional connectivity, global metrics in the hemispheric asymmetry were not significantly affected by tea drinking. This observation was in accordance with a previous finding of less overall alterations in functional connectivity relative to structural connectivity [33]. These results together allow speculation that structural global metrics are more sensitive to subtle alterations in the brain compared to functional global metrics in terms of overall connectivity at the network scale. This might not be the case for individual connections.

The Default mode network (DMN) functional connectivity has been extensively investigated as it related to the neurodegenerative brain [31, 32, 47–49]. A general finding is that suppressed activity and decreased functional connectivity in the DMN during rest is associated with cognitive decline [48–50]. Decreased functional connectivity linked to cognitive decline can be hindered or mitigated by tea intake according to the observation of stronger functional connectivity in the tea-drinking group. As is known, regions in the DMN are consistently found to be active and interconnected during the resting state [51] and engage in the preparation of a task implementation [52]. Stronger functional connectivity between regions of the DMN is related to better preparedness for task implementation. Therefore, the stronger functional connectivity in the DMN observed in the tea-drinking group may reflect the contribution of tea consumption to efficient network organization. Unlike functional connectivity, effects in structural connectivity in the DMN were of differing directions, with increasing connective strengths in some connections, but decreasing strengths for the others. We speculate that the coexistence of increased and decreased structural connectivity might be attributed to new, alternative paths established to replace disruption to existing routes.

Prior to our study, only two studies attempted to discover the relationship between tea intake and functional connectivity [29, 30]. Schmidt and colleagues found a significant increase in the functional connectivity strength between the right superior parietal lobule (SPL) and the right middle frontal gyrus (MFG) due to tea consumption [30]. The increase of functional connectivity strength was also observed in a comparative study [29]. Our study corroborated the previous notion that tea intake enhanced the strengths of specific functional connections and provided further insight that tea intake did not lead to a significant change in overall functional connectivity. In contrast to these two studies that focused on a few task-related brain regions for functional connectivity, our study investigated both global and regional functional connectivity and further elucidated the relationship between structural connectivity and tea drinking. Furthermore, these studies used a within-subject, short-term, tea intervention design [29, 30], whilst the current study performed a cross-sessional investigation to complement their studies.

Although our study was comprehensive and the findings were intriguing, the following limitations and considerations should be noted. The number of participants in our study was almost twice the numbers employed previously [29, 30]. However, the sample size was still limited. This was partially due to strict inclusion criteria which ensured that the participants in the tea drinking group had a habit of frequently drinking tea, while the participants in the non-tea drinking rarely or never drunk any kinds of tea to enhance the confidence of the findings. In our case, we initially recruited 93 participants, but only 15 and 21 participants remained in the tea-drinking group and non-tea drinking group, respectively.

Other substances might interfere with the outcome of the effect of tea drinking on brain connectivity. Coffee is one of the beverages that affect outcomes as it contains caffeine that is also one of the main active constituents of tea. However, coffee consumption between the tea-drinking group and the non-tea drinking group was not significantly different between the groups. Although there were no significant differences in demographic factors, environmental factors could have a confounding effect on brain network properties. For the nature of exploratory purpose, p-values were not corrected in the comparisons of regional properties between the tea drinking and non-tea drinking groups. Therefore, some of detected regions or connections may have occurred by chance. The results of regional properties provide heuristic information for further studies.

In summary, our study comprehensively investigated the effects of tea drinking on brain connectivity at both global and regional scales using multi-modal imaging data (i.e., functional and structural imaging) and provided the first compelling evidence that tea drinking positively contributes to brain structure making network organization more efficient. Our study suggests that tea drinking is effective in preventing (slowing) or ameliorating cognitive decline and that tea drinking might be a simple lifestyle choice that benefits brain health.

## MATERIALS AND METHODS

### Participants

Older participants were recruited from residential communities in Singapore by door-to-door visits. Trained research nurses and psychologists assessed participants to exclude those who had one or more conditions including dementia (Clinical Dementia Rating (CDR) global score>0), terminal illness, stroke, aphasia, marked hearing impairment, or any psychiatric or psychological problems. A set of neuropsychological and cognitive measures comprising the Rey Auditory Verbal Learning Test (RAVLT) [53], the Digit Span and Block Design tests from the Wechsler Adults Intelligence Scale (WAIS-III) [54], the Symbol Digit Modalities Test (SDMT) [55], the Boston Naming Test [56], the Color Trials Test 1 & 2 (CTT1 & CTT2) [57], the Singapore modified Mini-Mental State Examination (MMSE) [58, 59], and the Montreal Cognitive Assessment (MoCA) [60] were administered to participants. In this first wave of recruitment, a total of 93 participants attended the assessment and were subsequently recruited to a separate randomized controlled intervention trial in Singapore [61].

Recruited participants were stratified into two groups according to a composite score of tea drinking frequency, resulting in tea drinking and non-tea drinking groups. Drinking frequencies of green tea, oolong tea, black tea, and coffee were collected on a categorical scale ranging from 1 to 6 (see Table 2). Participants were required to recall their tea drinking habits, completing a questionnaire with drinking frequencies at age around 45 years and at present. The frequency scales of the three kinds of tea were first summed up separately for age around 45 years and the present, and then averaged across ages to obtain the composite score. If a participant had the composite score greater than or equal to 8, he/she was assigned to the tea drinking group. For example, a composite scale of 8 means that either two kinds of tea were simultaneously drunk at least 1-3 times a week, or one kind of tea was drunk 4-6 times a week or more, on average. Participants with the composite score of 3 (i.e. rarely or never drunk any kinds of tea) were assigned to the non-tea drinking group. This screening results in 15 participants in the tea drinking group and 21 participants in the non-tea drinking group. Demographic information is shown in Table 1.

**Table 2:** The scale of tea drinking frequency.

**Scale**	**Description**
1	Never or rarely
2	More than once a month but less than once a week
3	1-3 times a week
4	4-6 times a week
5	1-2 times a day
6	Greater than or equal to 3 times a day

The protocol was reviewed and approved by the Institutional Review Board of the National University of Singapore. Written, informed consent to participate in the study was provided by participants after they were given detailed information on the study.

### MRI data acquisition

MRI data were acquired by a 32-channel head coil on a 3T Prisma Siemens scanner (Siemens, Erlangen, Germany) located at the Clinical Imaging Research Centre (CIRC) of the National University of Singapore. T1-weighted images were acquired using a 3D magnetization-prepared rapid gradient-echo sequence with the parameters: repetition time [TR] = 2300 ms, echo time [TE] = 2.03 ms; field of view [FOV] = 256 × 256 mm^2^; slice thickness = 1mm, 176 slices, flip angle = 9°; acquisition matrix = 256 × 256; in-plane resolution = 1 × 1 mm^2^, which were utilized for co-registration, normalization, and cortical parcellation during data preprocessing.

Diffusion encoded images were acquired using a single-shot echo-planar sequence with 63 2-mm thick slices with no gap, TR = 8500 ms, TE = 96 ms, FOV = 192 × 192 mm^2^, acquisition matrix = 96 × 96; in-plane resolution = 2 × 2 mm^2^, b-value = [350 650 1000 1300 1600] s/mm^2^; 1 baseline image with b0 = 0 s/mm^2^. Each high b-value was acquired from 12 separate nonparallel diffusion directions.

Two hundred and ten (210) fMRI scans were obtained whilst participants rested with their eyes closed using the following echo-planar pulse sequence parameters: TR = 2550 ms, TE = 30 ms, FOV = 192 × 192 mm^2^, 42 slices, slice thickness = 3 mm, flip angle = 78°, acquisition matrix = 64 × 64, in-plane resolution = 3 × 3 mm^2^. These data were collected in a single scanning session. During scanning, earplugs were used to reduce the effect of scanner noise on participants and foam pads were used to minimize head motion.

### Data processing

Functional MRI was preprocessed by initially excluding the first ten volumes, followed by slice timing correction, head motion correction, local of the parenchyma, signal regression (including regressors of 24 head-motion parameters, and time-series in cerebrospinal fluid and white matter), spatial normalization to the Montreal Neurological Institute (MNI) space using the T1-weighted image, spatial smoothing with a 4.5-mm full width half-maximum Gaussian kernel, and temporal band-pass filtering (0.01 Hz ~ 0.1 Hz) to mitigate the effects of low frequency drift and high frequency physiological noise. To eliminate the interference of large head motions, participants were excluded if the maximal inter-scan motion exceeded 2 mm translation or 2 degrees rotation in any direction or the average frame-wise displacement (FD) exceeded 1 mm in the residual effect of head motion, according to the root mean square of FD [62]. With these criteria, no participant was excluded. Preprocessing was carried out using the statistical parametric mapping (SPM12 http://www.fil.ion.ucl.ac.uk/spm/software/spm12/), resting-state fMRI data analysis toolkit (REST) [63], and data processing assistant for resting-state fMRI (DPARSF) [64].

Diffusion-weighted images were realigned and corrected for head motion and eddy current distortions [62]. A diffusion tensor was fitted to each voxel within the brain parenchyma. A widely used deterministic streamline tracking algorithm was then utilized to identify white matter tracts, with the tracking starting from the deep white matter regions and terminating at a voxel either due to a turning angle >45º or a fractional anisotropy <0.15. The preprocessing of diffusion-weighted images was performed using the functional magnetic resonance imaging of the brain (FMRIB) Software Library in Version 5.0 (FSL, [65]), diffusion toolkit [66], and PANDA [67].

A previously validated Automated Anatomical Labeling (AAL) template [68] was employed to parcellate the cerebrum into 90 regions of interest (ROIs). We applied the same parcellation to both functional and diffusion data to enable direct comparison. Functional connectivity strength between a pair of ROIs was obtained by calculating the Pearson correlation coefficient between time series derived from averaging the time series of each voxel within each ROI. Structural connectivity strength between pairs of ROIs was computed as the number of streamlines normalized by the volumes of the two ROIs to eliminate bias due to different sizes [69]. Connectivity strengths of all possible pairs of ROIs were collected to construct a connectivity network, resulting in one functional connectivity network and one structural connectivity network for each participant.

### Data analysis

Graph theoretical analysis is a powerful and quantitative method to evaluate the properties of connectivity networks and has been being widely used in brain imaging studies. In this study, we explored both global and regional properties of functional and structural connectivity networks. The properties of local clustering and integration of connectivity networks were ascertained by the metrics of weighted clustering coefficient (C_w_) and characteristic path length (L_w_) [70, 71]. Local efficiency (E_loc_) and global efficiency (E_glob_) were utilized to characterize connectivity network efficiency [72], reflecting the efficiency of brain organization. A regional property was evaluated with the nodal efficiency [73], assessing the information transmission capability of an ROI.

To remove spurious connections from functional networks, a sparsity threshold was applied to only retain the connections with connective strengths above the threshold. Since there is no definitive method to determine the sparsity threshold [73], we calculated each network metric for the entire network (size: 90×90) at a series of thresholds ranging from 0.1 to 0.4 with an incremental step of 0.01, and then took the integral over these values to obtain a final quantity that was tested for between-group differences.

In the exploration of hemispheric asymmetry, inter-hemispheric connections were excluded and intra-hemispheric connections were divided into two connectivity sub-networks (size: 45×45) corresponding to the left and right hemispheres. The metrics were separately computed for the two sub-networks, X(L) and X®, for left and right hemispheres respectively [33] and the hemispheric asymmetry of a metric was then obtained by: [X(L) – X®] / [X(L) + X®], and then compared between groups. A positive value in the hemispheric asymmetry indicates the leftward hemispheric asymmetry while a negative value indicates the rightward hemispheric asymmetry.

We selected AAL atlas-based regions which belong to the default mode network (DMN), identified by previous work [74]: SFGdor.L, SFGdor.R, SFGmed.L, SFGmed.R, ACG.L, ACG.R, PCG.L, PCG.R, PHG.L, PHG.R, ANG.L, ANG.R, MTG.R, and ITG.L. Full names of the regions can be found in the Supplementary Table 1. Connectivity strengths amongst these selected regions were compared between the tea drinking and non-tea drinking groups.

All statistical evaluations in this study were performed by non-parametric permutation tests [75] unless otherwise stated. Specifically, the null hypothesis was that there was no difference in a variable between the tea-drinking group and the non-tea drinking group. P-values were obtained from null distributions estimated from 10,000 permutations of group labels. The threshold for significance was set a p<0.05 after the false discovery rate (FDR) correction (adopted the Benjamini and Hochberg procedure [76]) for the comparisons of global properties and neuropsychological measures. P-values were not corrected in the comparisons of regional properties for the exploratory purposes.

## Supplementary Material

Supplementary Table
