# Supplementary material for: Habitual tea drinking modulates brain efficiency: evidence from brain connectivity evaluation
Source: Aging (Albany NY). 2019 Jun 14;11(11):3876–90. doi: 10.18632/aging.102023 (PMC6594801; doi:10.18632/aging.102023)
Supplement: Supplementary Table [file aging-11-102023-s001.pdf]

SUPPLEMENTARY TABLE

Supplementary Table 1. The list of region names and their abbreviations of the default mode network based on the parcellation of the automated anatomical labeling (AAL) atlas.

Region name	Abbr.	Belongs to
Left superior frontal gyrus (dorsal)	SFGdor.L	Frontal
Right superior frontal gyrus (dorsal)	SFGdor.R	Frontal
Left superior frontal gyrus (medial)	SFGmed.L	Frontal
Right Superior frontal gyrus (medial)	SFGmed.R	Frontal
Left anterior cingulate and paracingulate gyri	ACG.L	Limbic
Right anterior cingulate and paracingulate gyri	ACG.R	Limbic
Left posterior cingulate gyrus	PCG.L	Limbic
Right posterior cingulate gyrus	PCG.R	Limbic
Left parahippocampal gyrus	PHG.L	Limbic
Right parahippocampal gyrus	PHG.R	Limbic
Left angular gyrus	ANG.L	Parietal
Right angular gyrus	ANG.R	Parietal
Right middle temporal gyrus	MTG.R	Temporal
Left inferior temporal gyrus	ITG.L	Temporal
